# SUNA and red ear syndrome: a new association and pathophysiological considerations

**DOI:** 10.1186/1129-2377-14-32

**Published:** 2013-04-08

**Authors:** Giorgio Lambru, Norazah Abu Bakar, Manjit Matharu

**Affiliations:** 1 Headache Group, Institute of Neurology and The National Hospital for Neurology and Neurosurgery, Queen Square, London, WC1N 3BG, UK; 2 The National Hospital for Neurology and Neurosurgery, Queen Square, London, UK

**Keywords:** SUNA, SUNCT, Red ear syndrome, Trigeminal autonomic cephalalgias, Cranial autonomic symptoms

## Abstract

Red ear syndrome (RES) is characterised by attacks of unilateral or bilateral burning ear pain associated with erythema. Primary and secondary forms have been described. Primary RES appears to have a frequent association with primary headaches especially migraine. Here, we describe the case of a woman with short-lasting unilateral neuralgiform attacks with cranial autonomic symptoms (SUNA) and recurrent episodes of ipsilateral red ear triggerable by cutaneous stimulation. Lamotrigine was beneficial for her SUNA but not for the RES. Both these disorders are extremely rare therefore their coexistence in the same individual may suggest similar pathophysiological mechanisms rather than a chance association.

## Background

Red ear syndrome (RES) is a rare condition originally described by Lance [1]. Since this initial description approximately 100 cases have been reported in the literature [2-5]. RES consists of unilateral or bilateral paroxysmal attacks of burning sensation and reddening of the external ear. The duration of these episodes ranges from seconds to hours, and the frequency can vary from a few monthly episodes to several daily attacks. Attacks can be spontaneous or provoked by cutaneous stimulation and changes in temperature. RES can be classified into primary or secondary forms with the latter often being associated with upper cervical spine diseases or temporomandibular joint dysfunction [2]. By definition, primary RES is not associated with any pathological disorders, although an association with primary headache syndromes including migraine [3] and paroxysmal hemicrania (PH) [6,7], has been reported.

Short-lasting unilateral neuralgiform headache attacks with conjunctival injection and tearing (SUNCT) is a rare primary headache disorder. The International Classification of the Headache Disorders (ICHD-2) diagnostic criteria for SUNCT require unilateral orbital, supraorbital or temporal, stabbing or pulsating attacks lasting from 5 to 240 seconds accompanied by ipsilateral conjunctival injection and lacrimation with a frequency ranging from 3 to 200 per day. According to the International Headache Society (IHS) classification committee, SUNCT may be a subform of a broader condition called short-lasting unilateral neuralgiform headache attacks with cranial autonomic symptoms (SUNA) where the above conditions must be met apart from only one cranial autonomic symptom needing to be present for diagnosis [8]. To date, SUNA is still poorly characterised with few cases described in the literature [9].

We provide the first description of SUNA occurring in association with RES and discuss the possible pathophysiological implications of this association.

## Case report

A 64 year-old woman was referred to our clinic with a five year history of facial pain. She had been having 40–200 severe daily attacks all of which were strictly unilateral, stabbing, and centred over the right cheek with radiation to the right upper gum and eye. Each episode would last one to two minutes and was associated with ipsilateral lacrimation, eyelid oedema, nasal blockage and facial sweating. Triggers included wind or cutaneous stimulation over her face, cleaning her teeth, eating, drinking and brushing her hair. There was no refractory period following an attack.

**Figure 1:**
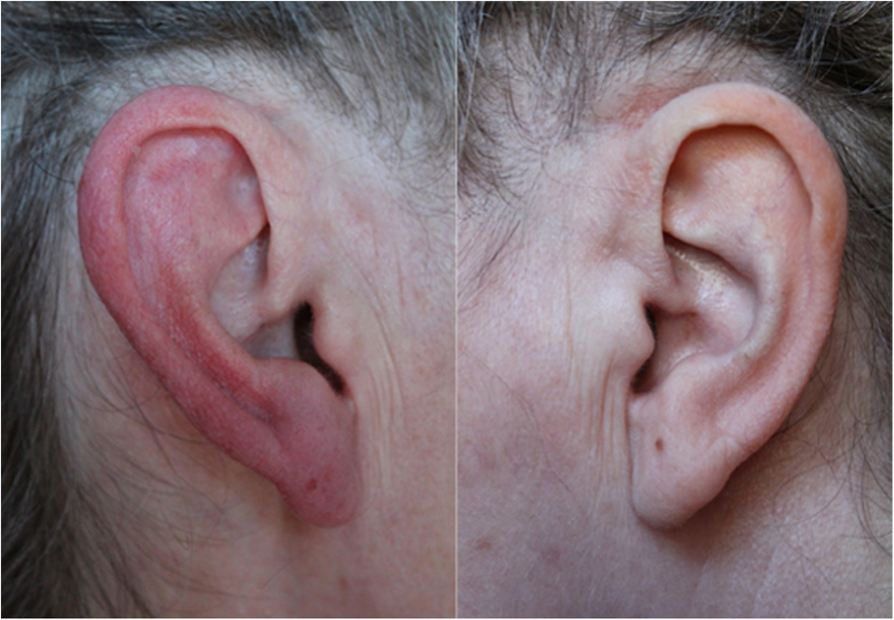
A red ear syndrome attack, provoked by rubbing the right ear.

She also complained of very different episodes consisting of marked reddening of her right ear associated with a moderate burning pain, started approximately at the time the stabbing attacks began. She had 5–10 episodes per month each lasting approximately 20–30 minutes each. These attacks occurred spontaneously but could also be triggered by gentle rubbing of the right ear for a few seconds (Figure 1). She reported that the stabbing attacks could also trigger these episodes of ear pain.

A general neurological examination and an MRI of the brain and the cervical spine were unremarkable with no vascular loops in contact with the trigeminal nerves. A diagnosis of chronic SUNA and primary RES was made and the patient placed on lamotrigine, titrating the dose by 25–50 mg every fortnight, up to 400 mg daily. This regime reduced her SUNA attacks down to 20–80 a day, according to the patient’s headache diaries, but had no discernible effect on the RES episodes. Trials of nortriptyline 50 mg, gabapentin 1800 mg and topiramate 75 mg in the past, had failed to provide any improvement either of the SUNA attacks or of the RES episodes.

## Discussion

To the best of our knowledge this is the first reported association between SUNA and RES. In this patient, both conditions affected the same side of the head, shared similar triggers, with painful attacks that could occur concurrently, although, given the high daily frequency of SUNA attacks, this may just have been a chance association. SUNA and RES are extremely rare conditions. The coexistence of these syndromes in an individual raises the possibility of co-morbidity rather than just mere association by chance. This leads us to speculate about a possible pathophysiological link between the two.

The pathophysiology of SUNCT and SUNA is poorly understood. Like the other trigeminal autonomic cephalalgias (TACs), abnormal activation of the trigeminal-autonomic reflex has been proposed to explain some of the clinical characteristics of SUNCT and SUNA syndromes, namely the trigeminal distribution of pain and the ipsilateral autonomic symptoms. The prominence of cranial autonomic symptoms in SUNCT and SUNA is thought to be caused by central disinhibition of this reflex possibly secondary to posterior hypothalamic dysfunction [10]. However, the considerable overlap in the clinical phenotypes of SUNCT, SUNA and trigeminal neuralgia (TN) along with the high proportion of ipsilateral vascular loops in contact with the trigeminal nerve recently reported in SUNCT and SUNA [11], has raised the possibility that additional peripheral mechanisms may have a role in their pathophysiology.

To understand the possible pathophysiological constructs for RES it is important to appreciate the neural and blood supply of the external ear. The sensory innervation of the earlobe and the lower external aspect of the ear are provided by the greater auricular nerve, a superficial branch of the C2-C3 spinal nerves, whereas the tragus and the anterosuperior aspect of the ear are innervated by the auriculotemporal branch of the mandibular division of the trigeminal nerve. The vasomotor control of the skin of the ear is regulated by variations in the vasoconstrictor tone rather than an activation of parasympathetic vasodilator fibres [12].

Originally, two theories involving peripheral mechanisms were proposed. An antidromic discharge of impulses along the C3 spinal nerve in response to the irritation provoked by the underlying lesion was the mechanism postulated for RES secondary to upper cervical pathologies, which account for most of the secondary forms of this disorder. To explain primary RES with spontaneous episodes and episodes triggered by mechanical or thermal stimulation, activation of an axon reflex with antidromic discharge of impulses in afferent C fibres and vasodilatation mediated via the discharge of vasodilator peptides (substance P, CGRP, nitric oxide) was proposed [2].

Subsequently, given the frequent association between primary RES and migraine as well as the co-occurrence of both migraine and RES episodes in individuals, it has been suggested that RES may be a migraine-related phenomenon caused by the activation of the trigeminovascular system, secondary to an intrinsic dysregulation of the brainstem trigemino-autonomic circuit [3].

Primary RES has also been described in association with TACs. Boes *et al.* reported a case of chronic paroxysmal hemicrania (CPH) characterized by attacks of left ear pain and redness associated with aural fullness. The attacks all lasted 2–3 minutes and occurred five times a day for at least 75% of the month. The pain subsided with a trial of indometacin 75 mg a day [6]. Subsequently, a case of RES associated with widespread facial/head pain and cranial autonomic features that resembled clinical features of cluster headache (CH) has been reported. The patient had episodes of ear pain and erythema, with the pain also perceived over the temple, cheek and upper neck and reddening that was associated with ipsilateral conjunctival injection, tearing and nasal blockage. Attacks occurred 1–6 times a day during the bouts [13]. The overlapping clinical characteristics between the majority of RES cases and TACs, namely unilateral nature, short-lasting duration and presence of autonomic features, points towards a possible pathophysiological link between the these disorders, with activation of the trigemino/cervical-autonomic reflex perhaps acting as the common final pathway for both the conditions [3]. Prior to our case, no association between RES and either SUNCT or SUNA in the same individual has been described. A previous paper has reported an apparent association between RES and SUNCT, but, in fact, actually appears to describe three independent cases of SUNCT, RES and hemicrania continua with no mention of co-existence of any of these syndromes in the same individual patient [14].

The co-existence of CH, CPH and SUNA, alongside RES may clinically suggest that the latter could be considered part of the TAC spectrum. This assumption could be supported by the speculation that the activation of the facial parasympathetic outflow that seems to account for most of the cranial autonomic features during TACs attacks might also produce ear skin vasodilation during RE episodes. However, unlike other parts of the face, such as the cheek and the nose, vasodilation of the skin of the ear is mainly under sympathetic vasoconstrictor control with parasympathetic fibres having a marginal role only [12]. Consequently, ear skin vasodilation seems to be caused by inhibition of sympathetic vasoconstriction fibres rather than activation of parasympathetic vasodilation fibres. It is known that in some CH patients there is a dysfunction in the cervical sympathetic pathway during an attack which leads to a disturbance in sympathetic vasoconstrictor tone and causes the classic features of ptosis and miosis [15]. If a similar process were to occur in RES one could speculate that this could cause disruption of sympathetic tone of the ear vessels resulting in vasodilation and reddening of the ear.

## Conclusion

In conclusion, the co-existence of SUNA and RES in the same individual supports the theory that the clinical entity of primary RES may actually be more suitably considered a form of TAC or maybe an auricular autonomic cephalagia as proposed by Lance [16]. The common clinical features in patients such as ours with both TAC and RES hint towards a common pathophysiological mechanism underlying the two such as an aberrant activation of the trigeminal-autonomic reflex along with an associated derangement of the cervical sympathetic system leading to the mixture of seemingly sympathetic and parasympathic clinical signs. However, given the fact that RES is also described in the absence of any other primary headache syndromes [17,18] is conceivable that a different mechanism, perhaps a local dysfunction of small sensory and sympathetic fibres of the ear, may play an important role in the pathophysiology of isolated cases of RES.

## Consent

Written informed consent was obtained from the patient for publication of this report and accompanying images.

## Competing interests

There are no competing interests. GL and NAB have no disclosures. MSM serves on the advisory board for Allergan and St Jude Medical, and has received payment for the development of educational presentations from Allergan, Merck Sharpe and Dohme Ltd and Medtronic. This work was undertaken at UCL/UCLH and was funded in part by the Department of Health NIHR Biomedical Research Centres funding scheme.

## Authors’ contributions

GL and NAB participated in the patient’s care, data collection and manuscript writing. MM participated in the patient’s care, data interpretation and revising the manuscript. All authors read and approved the final manuscript.
